# Leprosy among Patient Contacts: A Multilevel Study of Risk Factors

**DOI:** 10.1371/journal.pntd.0001013

**Published:** 2011-03-15

**Authors:** Anna M. Sales, Antonio Ponce de Leon, Nádia C. Düppre, Mariana A. Hacker, José Augusto C. Nery, Euzenir N. Sarno, Maria L. F. Penna

**Affiliations:** 1 Leprosy Laboratory, Oswaldo Cruz Institute (FIOCRUZ), Rio de Janeiro, Brazil; 2 Department of Epidemiology, Institute of Social Medicine, University of Rio de Janeiro, Rio de Janeiro, Brazil; London School of Hygiene and Tropical Medicine, United Kingdom

## Abstract

**Background:**

This study aimed to evaluate the risk factors associated with developing leprosy among the contacts of newly-diagnosed leprosy patients.

**Methodology/Principal Findings:**

A total of 6,158 contacts and 1,201 leprosy patients of the cohort who were diagnosed and treated at the Leprosy Laboratory of Fiocruz from 1987 to 2007 were included. The contact variables analyzed were sex; age; educational and income levels; blood relationship, if any, to the index case; household or non-household relationship; length of time of close association with the index case; receipt of bacillus Calmette-Guérin (BGG) vaccine and presence of BCG scar. Index cases variables included sex, age, educational level, family size, bacillary load, and disability grade. Multilevel logistic regression with random intercept was applied. Among the co-prevalent cases, the leprosy-related variables that remained associated with leprosy included type of household contact, [odds ratio (OR) = 1.33, 95% confidence interval (CI): 1.02, 1.73] and consanguinity with the index case, (OR = 1.89, 95% CI: 1.42–2.51). With respect to the index case variables, the factors associated with leprosy among contacts included up to 4 years of schooling and 4 to 10 years of schooling (OR = 2.72, 95% CI: 1.54–4.79 and 2.40, 95% CI: 1.30–4.42, respectively) and bacillary load, which increased the chance of leprosy among multibacillary contacts for those with a bacillary index of one to three and greater than three (OR = 1.79, 95% CI: 1.19–2.17 and OR: 4.07–95% CI: 2.73, 6.09), respectively. Among incident cases, household exposure was associated with leprosy (OR = 1.96, 95% CI: 1.29–2.98), compared with non-household exposure. Among the index case risk factors, an elevated bacillary load was the only variable associated with leprosy in the contacts.

**Conclusions/Significance:**

Biological and social factors appear to be associated with leprosy among co-prevalent cases, whereas the factors related to the infectious load and proximity with the index case were associated with leprosy that appeared in the incident cases during follow-up.

## Author Summary

Leprosy is an infectious disease that can lead to physical disabilities, social stigma, and great hardship. Transmitted from person to person, it is still endemic in developing countries, like Brazil and India. Effective treatment has been available since 1960, but early diagnosis of the disease remains the most effective way to stop the transmission chain and avoid late diagnoses and subsequent disabilities. Knowledge of the risk factors for leprosy can facilitate early detection; therefore, our study aimed to investigate the factors presented by leprosy patients and their contacts, who are considered at highest risk of contracting the disease. We studied 6,158 contacts of 1,201 patients under surveillance from 1987 to 2007 in a Public Health Care Center in the City of Rio de Janeiro, Brazil. We evaluated the ways patient and contact demographics and epidemiological characteristics were associated with the detection of leprosy. Statistical analyses took into account both individual and group characteristics and their interrelationships. The main characteristics facilitating the contraction of leprosy among contacts were shown to be consanguinity and household association. Conversely, the bacillary load index of leprosy patients was the principle factor leading to disease among their contacts.

## Introduction

The primary aim of all disease control measures is to reduce the incidence, prevalence, morbidity and/or mortality rates to the lowest level possible in a given population. However, once control program objectives have been met, continuous interventions are necessary to maintain these minimal rates [1].

In 2007, the Brazilian Ministry of Health adopted new case detection rates for all ages and for children under 15 years of age as indicators of the effectiveness of leprosy control measures in the country. Because detection of leprosy in those under 15 years of age is considered indicative of recent *Mycobacterium leprae* (ML) transmission, evaluating these cases for epidemiologic markers was especially important [2].

In early 2009, the global prevalence of leprosy was approximately 213,000 cases; however, the annual detection rate of leprosy worldwide has declined. In 2002, more than 620,000 cases were detected; whereas, in 2008, there were approximately 249,000 cases. In Brazil, in 2008, there were 38,914 new leprosy cases detected. Nevertheless, there seems to be a tendency for the detection rates to stabilize in Brazil at somewhat higher levels in the North, Midwest and Northeast regions of the country. In the state of Rio de Janeiro, there is a clear decreasing trend from 1990 to 2008. For instance, detection rates ranged from 27.30 cases per 100,000 population in 1997 to 11.84 cases per 100,000 population in 2008. The detection rates in Rio de Janeiro for children less than 15 years old in the period 2001–2008 had very high ratings (6.00/100,000 population to 2.69/100,000 population).

In addition to the administration of multidrug therapy (MDT) to patients diagnosed with leprosy, disease control strategies in Brazil include early new case detection, routine clinical examinations, and *Bacillus Calmette-Guérin* (BGG) vaccination of the patient's contacts, which is a group considered to be at high risk to develop the disease [3].

One activity of early detection of leprosy is contact surveillance, which aims to interrupt disease transmission and prevent the development of disabilities [4].

The notion that group-level factors are important in understanding the risk of disease has long been present in infectious disease epidemiology, because the risk of an individual contracting an infectious disease depends not only on his or her own risk behavior and biological and socio-economic factors, but also on his or her population group. With regard to scientific validity and the practical implications for disease prevention, the growing consensus is that investigations into the causes of disease must include factors defined on multiple levels, such as the individual and communities. In infectious disease epidemiology, multilevel analysis can be used to examine how both group- and individual-level factors are related to individual-level infectious disease outcomes and how factors on both levels affect group differences in the risk of disease. The application of multilevel analysis has only recently begun to emerge in the infectious disease literature [5], [6].

Several potential risk factors associated with individual features of leprosy patients and their contacts have been suggested but, to date, these factors' effects have yet to be evaluated. In-depth investigation of these factors may allow for the simultaneous examination of group-level and individual-level factors, assessment of the demonstrable interaction between contacts- and index case-level constructs, and exploration of how factors at multiple levels contribute to differences in disease risk.

The aim of the present study was to identify potential risk factors of the index cases and their contacts on development of leprosy among contacts.

## Materials and Methods

### Study population

Since 1987, the Leprosy Outpatient Clinic, a National Reference Center at the Oswaldo Cruz Foundation in Rio de Janeiro, RJ, Brazil, has conducted routine clinical examinations of the contacts of leprosy patients diagnosed at the Clinic. The Clinic provides health care recommendations to leprosy patients and their families at diagnosis and during treatment.

The study population consisted of 6,158 contacts of 1,201 newly-diagnosed leprosy patients of the cohort treated at the Leprosy Outpatient Clinic from 1987 to 2007. The average duration of follow-up of contacts was 16.9 years. Among the patients, 454 had paucibacillary leprosy, and 747 had multibacillary leprosy.

After confirmation of the leprosy diagnosis, patients were given educational information about the disease, and medical visits were scheduled for their close contacts (within and outside of the household). During the initial visits, contacts answered a questionnaire regarding socio-economic status (income and education level) and type of contact with the index case. The contacts were examined by specialized dermatologists and neurologists to confirm a leprosy diagnosis and the existence of a BCG scar.

The Brazilian Ministry of Health recommends that all leprosy contacts receive the BCG vaccine [3]. Between 1987 and 1991, all contacts were instructed to attend the Clinic at least once a year. From January 1992 throughout December 2007, they were also requested to return to the Clinic if and when symptoms and/or skin lesions appeared. Follow-up visits included medical consultations with specialized dermatologists and neurologists. Those presenting signs or symptoms that were suggestive of leprosy were assessed through bacteriological, histopathological, and immunological examinations.

In September 2009, the Brazilian Disease Notification System (SINAN), covering December 1987 to September 2009, was searched to locate the healthy contacts to ascertain whether any leprosy cases had been missed in contact follow-up procedures. SINAN records were matched to the database of the study group with respect to the variables present in both: name of contact, date of birth and mother's full name. Contacts that had not been identified as leprosy patients in SINAN by September 2009 were considered healthy.

Co-prevalent cases were the contacts diagnosed with leprosy at the first examination after the index case was diagnosed. Incident cases were apparently leprosy-free contacts at the time of index case diagnosis but developed the disease at some point during follow-up.

Household contacts were defined as individuals who had lived in the same dwelling during the five-year period prior to the index case diagnosis. Non-household contacts were defined as those indicated by the index case as having had other types of contact, such as next-door neighbors, blood relatives, friends and/or co-workers, etc., during the five-year period prior to the index case diagnosis.

Variables that described the contact included sex; age; educational and income levels; blood relationship, if any, and type (household and non-household) and length of time of close association with the index case. With regard to BCG vaccination, contacts were examined to verify the presence or absence of a BCG scar, which was considered the first dose. Once a leprosy diagnosis is excluded, the BCG vaccine is administered to a healthy contact, and this vaccination corresponded to the second dose. For the index cases, the variables included sex, age, educational level, family size, bacillary index (BI) from the slit skin smear test at the beginning of treatment and disability grade.

The patients were classified as paucibacillary, based on a zero BI, or multibacillary, based on an above-zero BI.

We classified the initial disability/impairment grade according to the present World Health Organization classification system [7], which consisted of three grades (0, 1 and 2). Grade 0 indicates no loss of sensation or visible deformity, grade 1 is defined by the loss of sensation without visible deformity, and grade 2 indicates the presence of a visible deformity. All disability grade evaluations were conducted by specialized professionals.

### Statistical analysis

A two-level logistic model with a random intercept was used, and the contacts were considered first-level units and grouped with their respective index cases, who were considered second-level units. For the empty models, the Variance Partition Coefficient (VPC) was calculated according to the simulation method proposed by Goldstein et al [8]. The total number of simulations was 5,000.

Initially, a bivariate analysis was conducted separately for the co-prevalent and incident cases. The association between the occurrence of leprosy disease and a set of independent variables was assessed using the crude odds ratio (OR) and the associated 95% confidence interval (CI).

The second step of the analysis involved adjusting the multilevel logistic regression model for all the contact and index case variables (full model – data not shown.)

The final model consisted of all the variables that were statistically significant after adjustment for all other factors related to the contacts and their respective index cases. Additional variables in the final included those recognized for epidemiological relevance or were frequently regarded as confounding variables, such as age of contact, sex, and contact and index case educational levels.

The estimated measure of association was the OR. The OR associated with incident-case risk factors may be interpreted as a relative risk (RR) when the disease frequency is low, as in the present study. The OR of prevalence cases also estimates the RR if the disease duration among the exposed and unexposed is the same [9].

The software MlWin 2.10 was used to perform the multilevel statistical analysis. The estimation method of Penalized Quasi-Likelihood, second order, was adopted throughout the analysis.

All contacts who returned to the clinic for examination were eligible for the study. All adult participants and the guardians or parents of the children that were included in the study provided written consent. This study was approved by the Ethics Research of the National School of Public Health.

## Results

This study included 6,158 contacts of 1,201 leprosy patients, with an average 5.12 contacts per patient. Of the contacts studied, 57.6% (3546/6158) were female. The mean age was 25.6 (±17,8) years. Of the index cases, 63.9% (767/1201) were male, and the mean age was 38.2 (±16,9) years.

Among the contacts, 452 (7.3%) new cases of leprosy were diagnosed. The first contact examination found 319 (5.2%) co-prevalent cases, and during the follow-up, 133 (2.3%) incident cases were diagnosed. Among the incident cases, this study found an incidence rate of 3.32 cases per person-year. The average period for the incident cases of leprosy diagnosis was 4.1 years after the index case diagnosis.

Among the contacts diagnosed with leprosy, 89.4% (404/452) had multibacillary leprosy, 74.5% (337/452) had paucibacillary leprosy, and 65.8% of them (222/337) had *borderline-tuberculoid* leprosy.

Table 1 shows the numbers and proportion of contacts with leprosy according to the clinical classification of index cases.

**Table 1:** New cases of leprosy among contacts according to type of index case.

Type of index case leprosy	Contacts with leprosy	Co-prevalent cases	Proportion(95% CI)	Incident cases	Proportion(95% CI)	Total of contacts(n)
Paucibacillary(n = 454)	48	39	2,4 (1,61–2,98)	9	0,5 (0,17–0,82)	1665
Multibacillary(n = 747)	404	280	6,3 (5,48–6,92)	124	2,9 (2,30–3,29)	4493

Abbreviations: CI, confidence interval.

The VPCs were approximately 18% and 13% for the co-prevalent and incident cases, respectively, i.e., the proportion of the outcome variability due to the determinants on the first level was somewhat greater in incident patients than in co-prevalent patients.

The frequencies and the bivariate analyses for the contacts and index cases, for the co-prevalent and incident cases are shown in Table S1.

A significant association was observed between the contacts diagnosed with leprosy at the initial examination (co-prevalent cases) and several of the variables under study; these included few years of schooling (OR = 1.50, 95% CI: 1.03–2.19), a monthly family income under three minimum wages (OR = 1.85, 95% CI: 1.35–2.54 and OR = 2.18 95% CI: 1.50–3.17), consanguineous relationship with (OR = 1.50, 95% CI: 1.15–1.96) and close proximity to the index case for a minimum five-year period (OR = 2.64, 95% CI: 1.75–3.98). Household contacts were more likely than non-household contacts to present with leprosy, for both co-prevalent cases (OR = 1.44, 95% CI: 1.11–1.86) and incident cases (OR = 2.05, 95% CI: 1.35–3.11). Having received a neonatal BCG vaccine was a protective factor in both co-prevalent and incident cases. In addition, the application of the BCG vaccine, as recommended by the Ministry of Health, was also a protective factor in the follow-up.

Among the index case variables, some were associated with a leprosy diagnosis in co-prevalent cases; these included up to 4 years of schooling (OR = 3.31, 95% CI: 1.87–5.58), between 4 to 10 years of schooling (OR = 2.53, 95% CI: 1.37–4.64), monthly family income up to two minimum wages (OR = 2.17, 95% CI: 1.34–3.52), having an income between two and three minimum wages (OR = 2.31, 95% CI: 1.44–3.70), and a disability grade = 2 (OR = 1.50, 95% CI: 1.04–2.16). The contacts who were 15 years and older had an increased odds ratio (OR = 8.37, 95% CI: 1.12–62.4) of contracting leprosy, compared with those who were under 15, only among incident cases. Contacts of male index cases were more likely to have leprosy than contacts of female index cases. This was true for both prevalent and incident leprosy cases among contacts. BIs of index cases over three was significantly associated with the diagnosis of co-prevalent leprosy cases (OR = 4.37, 95% CI: 2.95–6.46). BIs of one to three (OR = 4.30, 95% CI: 2.12–8.71) and more than three (OR = 7.31, 95% CI: 3.63–14.75) were associated with incident leprosy cases, considering as reference a negative BI.

Table 2 summarizes the results of the multivariate analysis. In the final model for co-prevalent cases, the variables that remained associated with leprosy between contacts were household contact (OR = 1.33: 95% CI: 1.02–1.73) and consanguinity with the index case (OR = 1.89, 95% CI: 1.42–2.51). With respect to the index case model, the variables associated with leprosy included up to 4 years of schooling and 4 to 10 years of schooling (OR = 2.72, 95% CI: 1.54–4.79 and 2.40, 95% CI: 1.30–4.42, respectively), and bacillary index, which increased the risk of leprosy among contacts for those with index cases with BI of one to three and greater than three (OR = 1.79, 95% CI: 1.19–2.70 and OR: 4.07, 95% CI: 2.73–6.09, respectively).

**Table 2:** Final model for contact and index case variables.

	Co-prevalent cases		Incident cases	
Contacts variables	a OR	95% CI	a OR	95% CI
***Age (years)***				
≥15	1		1	
<15	0.86	0.62–1.18	1.06	0.66–1.70
***Sex***				
female	1		1	
male	1.12	0.88–1.43	0.79	0.54–1.17
***Educational level (years)***				
>10	1		1	
4 to 10	1.08	0.61–1.94	0.40	0.16–1.01
<4	1.43	0.96–2.15	0.82	0.49–1.36
***Blood relationship***				
not blood related	1		1	
blood reated	1.89	1.42–2.51	1.54	1.00–2.37
***Type of close association***				
nonhousehold	1		1	
household	1.33	1.02–1.73	1.96	1.29–2.98
***BCG scar***				
no	1		1	
yes	0.28	0.21–0.37	0,45	0.30–0.68
***BCG vaccine***				
no	nd	nd	1	
yes	nd	nd	0.44	0.29–0.64
**Index cases variables**				
***Sex***				
female	1		1	
male	1.05	0.76–1.45	1.22	0.70–1.76
***Educational level (years)***				
<4	2.72	1.54–4.79	0.60	0.34–1.06
4 to 10	2.40	1.30–4.42	0.70	0.37–1.32
>10	1		1	
***Bacillary index***				
0	1		1	
1 to 3	1.79	1.19–2.70	4.64	2.26–9.55
>3	4.07	2.73–6.09	8.63	4.14–17.97

Abbreviations: a OR, adjusted odds ratio; BCG, *Bacillus Calmette-Guérin*; CI, confidence interval.

In the multilevel model for incident cases, household exposure was associated with leprosy in the incident case contacts, with OR = 1.96 (95% CI: 1.29–2.98). The consanguinous relationship of contacts with their index case was also a significant risk factor for contracting leprosy (OR = l.54, 95% CI: 1.00–2.37). In connection with index case variables, an elevated bacillary load was the only variable whose association was maintained after adjusting for the other variables under consideration.

The presence of a BCG scar showed a highly statistically significant protective effect in both models for co-prevalent and incident cases, with OR = 0.28 (95% CI: 0.21–0.37) and 0.45 (95% CI: 0.30–0.68), respectively. The contacts who received the BCG vaccine also demonstrated significant protection against the disease: OR = 0.44 (95% CI: 0.29–0.64).

There were no statistically significant differences in the odds between male and female contacts in either incident or co-prevalent cases.

Finally, the presence of overdispersion in the final models was not detected. The overdispersion parameter in the model for co-prevalent cases was 0.89 and that for incident cases was 0.94.

## Discussion

In this study, we found that the major risk factor among contact incident cases was proximity to the index case. Among the characteristics of the index cases, bacillary load was the only risk factor associated with developing leprosy. A BCG scar and the application of the vaccine after index case diagnosis independently contributed as protective factors. However, among co-prevalent cases, the variables most strongly associated were a consanguinous and household relationship with the index case. Furthermore, a BCG scar contributed independently as a protective factor. Factors related to the index cases included up to 4 years and between 4 to 10 years of schooling and bacillary load, both associated with leprosy among their contacts at the first examination.

Although men make up most of the leprosy cases in Brazil, our study did not find any gender differences in the risk of contracting the disease among contacts, suggesting that the gender differences in the detection rates for the general population may be due to differences in their exposure. These findings are in agreement with those of other studies that likewise did not observe any gender differences in the likelihood of acquiring leprosy [10]–[12] Nevertheless, Ali et al. [13], in a prospective contact study and two other retrospective studies, found that the attack rate was, in fact, lower among women [14], [15]. Conversely, Fine et al. [16] reported a significantly higher attack rate among men.

In the present study, contact age was not associated with leprosy among either co-prevalent or incident cases. Our decision to categorize the age of minors and those over 15 years to conform to the indicator adopted by the Brazilian Leprosy Control Program may be an explanation for this lack of association. Other studies have shown that among contacts the risk of leprosy is significantly higher for those younger than 14, particularly for contacts of multibacillary index cases [11], [13], [17]. Likewise, Moet et al. [12] reported a bimodal distribution according to age: the risk increased for those between 5 to 15 years of age, reached a peak for those aged 15 to 20, decreased for those aged 20 to 29, and gradually increased after a 30-year lag.

Leprosy has traditionally been associated with lower socio-economic status. An ecological study recently conducted in Brazil by Kerr et al. [17] showed an association between social inequality, population growth and a high prevalence of leprosy. Population-based studies have also described an increased risk of leprosy associated with fewer years in school, poor housing and low income [18]. Our findings suggested an association between level of education and leprosy. However, in our study, poor schooling was associated with disease duration in index case patients and with a higher prevalence of leprosy among their close contacts (co-prevalence). Poor schooling among index case patients is likely to be a proxy for lower socio-economic status and could be associated with late diagnosis of leprosy, allowing for longer periods of exposure among their contacts. This finding is most certainly related to both the unavailability and inaccessibility of health care facilities, making it more difficult for individuals to maintain good health and prevent disease. In the present study, the lack of association between socio-economic markers and the risk of disease could be understood in light of the homogenous distribution of these markers along the study sample; everyone involved in this study was from the same socio-economic strata.

From the moment of the index case diagnosis, the consanguinous relatives had a higher risk of developing leprosy (OR = 1.89, 95% CI: 1.42–2.51). Most likely due to their increased vulnerability, genetic susceptibility, and type of immune response, these contacts were more likely to become ill. In turn, the confidence interval of the probability of association with incidence cases was just above the cut-off probability of 0.05. A cross-sectional study on determinants of the transmission of leprosy showed that consanguinous relatives had a 2.8 higher risk than non-consanguineous contacts [19]. Similarly, Moet et al. [12], in the initial evaluation of a contact cohort, calculated that consanguinous contacts had an increased odds (OR = 1.65, 95% CI: 1.05, 2.57), regardless of physical distance from their index case.

As expected, contact/index case co-habitation was shown to be a key risk factor in developing leprosy. However, the strength of this association was different for both co-prevalent and incident cases. Household contacts had a higher risk for leprosy in the follow-up. Among incident cases, the risk of household contacts developing the disease was twice that among non-household contacts, which also corroborated findings of aforementioned studies. To reiterate, a number of reports have indicated that household contacts are at the highest risk, compared with the general population [11], [14], [20] and non-household contacts [16], [21].

As in other, similar studies, the most important association determining leprosy disease among contacts was the bacillary load of the index case. These findings were in agreement with the literature that demonstrates that multibacillary patients are primarily responsible for ML transmission in endemic areas [10]–[13], [15], [20], [22]. In the follow-up, index cases with BIs over three were eight times more likely to transmit leprosy to their contacts (incident cases) than were paucibacillary patients. The contacts of multibacillary index cases also had a four-fold higher chance of being diagnosed with leprosy (co-prevalent cases) than did the contacts of index cases with a negative BI. A previous study conducted in Brazil demonstrated that a high familial bacillary index and the presence of more than one source of contamination in the family at the time of first examination of contacts were associated with greater risk of developing leprosy, especially among those younger than 15 years [23].

Again, in the present study, the BCG vaccine administered in infancy was shown to effectively protect against leprosy in 72% [(1-OR)×100] of all co-prevalent cases and 55% of incident cases. During the follow-up, the protective rate conferred by the BCG vaccine applied after index case diagnosis was 56% [(1-OR)×100)]. Other Brazilian studies have confirmed the significant impact of neonatal BCG on the incidence and transmission of leprosy [24], [25]. In our study, of the contacts vaccinated who developed leprosy in the follow-up period, 89% have presented with the paucibacillary form of the disease, indicating the protective effect of BCG vaccine against the development of multibacillary forms, consistent with other studies that point to the role of vaccine in the interruption of leprosy transmission [26], [27].

In summary, socio-economic factors appear to be more strongly associated with leprosy among the contacts found to be ill at the first examination (co-prevalent cases), compared with the association among incident cases. This finding among co-prevalent cases may be secondary to the difficulties that patients with lower educational level have in finding adequate health care facilities and information. With regard to the incident cases, bacillary load factors, i.e., intensity of transmission, increased the likelihood of contracting leprosy, in comparison with other social and biological factors. Moreover, incident cases developed the disease even when the associated co-prevalent and index cases were undergoing treatment, had neurological and skin examinations, and received the BCG vaccination.

A major strength of this study was the multilevel approach in analyzing the data, which allowed for the simultaneous observation of the effects of the predictor variables on both the group (index case) and individual levels (contacts). Importantly, inter-group-dependent observations were taken into account, which highlighted and did not disregard the dependency of leprosy as an infectious disease. According to the evaluation of VPC in Goldstein [8], with regard to empty models, the leprosy variance among contacts that can be attributed to the differences among index cases was 18% of the co-prevalent and 13% of the incident cases. Moreover, we observed that outcome variability at the superior hierarchical level was sufficient to justify the use of this model. We also found that the VPC evaluation of the final models indicated that only 2.4% and 4.0% of the explained variables continued to be attributable to the index cases, whereas the model appears to be well fitted for both the co-prevalent and incident cases.

The ability to accurately identify contacts of leprosy patients who are at high risk of disease is of utmost importance for leprosy control. Surveillance and appropriate health education of household contacts should be strongly reinforced and extended to all close contacts of index case-patients, including their consanguineous relatives. In our study, however, we have identified a group of contacts who, despite all appropriate intervention measures, acquired leprosy. Therefore, household contacts of MB index case-patients, especially those with high bacillary load at diagnosis, should be considered for chemoprophylaxis in addition to immunoprophylaxis with BCG vaccination, once the efficacy of chemoprophylaxis is proven.

## Supporting Information

Table S1 Frequencies and the bivariate analyses for the contacts and index cases. Abbreviations: BCG, *Bacillus Calmette-Guérin*; CI, confidence interval; c OR, crude odds ratio.
(DOC)
