# Correction: Leprosy among Patient Contacts: A Multilevel Study of Risk Factors

**DOI:** 10.1371/annotation/fc90a4dd-99a7-4c48-9dee-0494a0180038

**Published:** 2011-08-12

**Authors:** Anna M. Sales, Antonio Ponce de Leon, Nádia C. Düppre, Mariana A. Hacker, José Augusto C. Nery, Euzenir N. Sarno, Maria L. F. Penna

The third paragraph of the Results section should read "Among the total number of contacts diagnosed with leprosy, 89.4% (404/452) had multibacillary index cases. Of the total contacts, 74.5% (337/452) had paucibacillary leprosy, 65% of whom (222/337) had the borderline-tuberculoid form."

