# Supplementary material for: Leprosy among Patient Contacts: A Multilevel Study of Risk Factors
Source: PLoS Negl Trop Dis. 2011 Mar 15;5(3):e1013. doi: 10.1371/journal.pntd.0001013 (PMC3057944; doi:10.1371/journal.pntd.0001013)
Supplement: Table S1 — Frequencies and the bivariate analyses for the contacts and index cases. Abbreviations: BCG, Bacillus Calmette-Guérin; CI, confidence interval; c OR, crude odds ratio. (DOC) [file pntd.0001013.s001.doc]

**Table S1. Frequencies and the bivariate analyses for the contacts and index cases.**

|  |  |  | **Co-prevalent cases** | | | **Incident cases** | | |
| --- | --- | --- | --- | --- | --- | --- | --- | --- |
| **Contacts Variables** | **total (n)** | **cases (%)** | **n** | **c OR** | **95% CI** | **n** | **c OR** | **95% CI** |
| ***Age (years)*** |  |  |  |  |  |  |  |  |
| ≥ 15 | 4108 | 8.0 | 236 | 1 |  | 92 | 1 |  |
| < 15 | 2050 | 6.0 | 83 | 0.65 | 0.50-.84 | 41 | 0.85 | 0.58-1.24 |
| ***Sex*** |  |  |  |  |  |  |  |  |
| female | 3546 | 7.4 | 177 | 1 |  | 84 | 1 |  |
| male | 2612 | 7.3 | 142 | 1.07 | 0.85-1.34 | 49 | 0.78 | 0.55-1.13 |
| ***Educational level (years)*** |  |  |  |  |  |  |  |  |
| > 10 | 1112 | 6 | 39 | 1 |  | 28 | 1 |  |
| 4 to 10 | 718 | 4.5 | 25 | 0.93 | 0.53-1.65 | 7 | 0.38 | 0.15-0.94 |
| < 4 | 4327 | 8.2 | 255 | 1.50 | 1.03-2.19 | 98 | 0.91 | 0.56-1.47 |
| ***Income level (minimum wages)*** |  |  |  |  |  |  |  |  |
| > 3 | 2561 | 4.9 | 87 | 1 |  | 39 | 1 |  |
| 2 to 3 | 2435 | 9.0 | 150 | 1.85 | 1.35-2.54 | 69 | 1.90 | 1.21-2.96 |
| < 2 | 1162 | 9.2 | 82 | 2.18 | 1.50-3.17 | 25 | 1.47 | 0.84-2.60 |
| ***Blood relationship*** |  |  |  |  |  |  |  |  |
| not blood related | 1951 | 5.5 | 75 | 1 |  | 33 | 1 |  |
| blood related | 4207 | 8.2 | 244 | 1.50 | 1.15-1.96 | 100 | 1.37 | 0.91-2.05 |
| ***Type of close association*** |  |  |  |  |  |  |  |  |
| nonhousehold | 2477 | 5.4 | 99 | 1 |  | 35 | 1 |  |
| household | 3681 | 8.6 | 220 | 1.44 | 1.11-1.86 | 98 | 2.05 | 1.35-3.11 |
| ***Lengh of time of close association (years)*** |  |  |  |  |  |  |  |  |
| < 5 | 1083 | 4.1 | 26 | 1 |  | 18 | 1 |  |
| ≥ 5 | 5075 | 8.0 | 293 | 2.64 | 1.75-3.98 | 115 | 1.47 | 0.87-2.48 |
| ***BCG scar*** |  |  |  |  |  |  |  |  |
| no | 2250 | 12.1 | 201 | 1 |  | 71 | 1 |  |
| yes | 3908 | 4.6 | 118 | 0.31 | 0.24-0.39 | 62 | 0.47 | 0.33-0.67 |
| ***BCG vaccine*** |  |  |  |  |  |  |  |  |
| no | 2189 | 17.4 | * | * | * | 67 | 1 |  |
| yes | 3969 | 1.8 | * | * | * | 66 | 0.44 | 0.30-0.64 |
| **Index Cases Variables** | | | | | | | | |
| ***Age (years)*** |  |  |  |  |  |  |  |  |
| < 15 | 358 | 5.0 | 17 | 1 |  | 1 | 1 |  |
| ≥ 15 | 5800 | 7.5 | 302 | 0.96 | 0.53-1.72 | 132 | 8.37 | 1.12-62.4 |
| ***Sex*** |  |  |  |  |  |  |  |  |
| female | 2149 | 5.9 | 94 | 1 |  | 32 | 1 |  |
| male | 4009 | 8.1 | 225 | 1.47 | 1.07-2.01 | 101 | 1.61 | 1.03-2.53 |
| ***Educational level (years)*** |  |  |  |  |  |  |  |  |
| > 10 | 850 | 4.8 | 17 | 1 |  | 24 | 1 |  |
| 4 to 10 | 1479 | 6.8 | 70 | 2.53 | 1.37-4.64 | 31 | 0.70 | 0.37-1.31 |
| < 4 | 3829 | 8.1 | 232 | 3.31 | 1.87-5.58 | 78 | 0.70 | 0.40-1.21 |
| ***Income level (minimum wages)*** |  |  |  |  |  |  |  |  |
| > 3 | 1491 | 4,3 | 40 | 1 |  | 24 | 1 |  |
| 2 to 3 | 1428 | 8,2 | 84 | 2.31 | 1.44-3.70 | 33 | 1.48 | 0.78-2.78 |
| < 2 | 1254 | 8,0 | 73 | 2.17 | 1.34-3.52 | 27 | 1.36 | 0.70-2.63 |
| ***Family size (persons)*** |  |  |  |  |  |  |  |  |
| < 5 | 2413 | 8,1 | 149 | 1 |  | 46 | 1 |  |
| ≥ 5 | 3745 | 6,9 | 179 | 0.71 | 0.53-0.95 | 87 | 1.19 | 0.79-1.79 |
| ***Bacillary index*** |  |  |  |  |  |  |  |  |
| 0 | 1822 | 3.0 | 44 | 1 |  | 10 | 1 |  |
| 1 a 3 | 2491 | 6.5 | 104 | 1.91 | 1.28-2.86 | 57 | 4.30 | 2.12-8.71 |
| > 3 | 1804 | 12.7 | 165 | 4.37 | 2.95-6.46 | 65 | 7.31 | 3.63-4.75 |
| ***Disability grade*** |  |  |  |  |  |  |  |  |
| 0 | 3270 | 6.5 | 153 | 1 |  | 61 | 1 |  |
| 1 | 1635 | 8.4 | 91 | 1.30 | 0.92-1.83 | 46 | 1.59 | 1.00-2.52 |
| 2 | 1253 | 8.1 | 75 | 1.50 | 1.04-2.16 | 26 | 1.13 | 0.66-1.93 |
